# The efficacy of electroacupuncture for cervical nerve edema and movement disorder caused by the brachial plexus injury: a case report

**DOI:** 10.3389/fneur.2024.1342844

**Published:** 2024-04-23

**Authors:** Chao Wang, Yingjun Liu, Lu Li, Haijuan Zhang, Ziyu Ye, Linfang Zhao

**Affiliations:** ^1^The Third Affiliated Hospital of Zhejiang Chinese Medical University, Hangzhou, China; ^2^Guangzhou University of Chinese Medicine, Guangzhou, China

**Keywords:** brachial plexus injury, electroacupuncture, cervical nerve edema, movement disorder, case report

## Abstract

The brachial plexus injury (BPI) is one of the most severe types of peripheral nerve injuries, often caused by upper limb traction injury. In clinic, the surgery is widely used to treat the BPI. However, surgery may need to be performed multiple times at different stages, which carries risks and brings heavy economic burden. In non-surgical treatment, splinting, local injection of corticosteroids, and oral corticosteroids can achieve significant short-term benefits, but they are prone to recurrence and may cause complications of mechanical or chemical nerve damage. In this report, we present a case of a 46-year-old female patient with BPI. The patient had difficulty in raising, flexing and extending of the left upper limb, and accompanied with the soreness and pain of neck and shoulder. After 3 months of EA treatment, a significant reduction in the inner diameter of the left C5 to C7 root at the outlet of brachial plexus nerve was detected by musculoskeletal ultrasound, and the soreness and pain in the left neck and shoulder were significantly reduced. The soreness and pain in the left neck and shoulder did not recur for 2 years.

**Case summary:**

The patient is a 46-year-old female with BPI. She experienced difficult in lifting, flexing and extending of the left upper limb, which accompanied by soreness and pain in the left neck and shoulder. After 3 months of EA treatment, the patient's pain and limb's movement disorder was improved. After 2 years of follow-up, the patient's left neck and shoulder showed no further pain.

**Conclusion:**

EA has shown satisfied efficacy in BPI, improving limb restrictions and relieving pain in patients for at least 2 years.

## Introduction

Brachial plexus injury (BPI) is one of the most common peripheral nerve injuries and is a disabling condition closely associated with trauma, fractures, and cervical spondylosis (1). The impairment of limb function significantly affects the patients' work and daily life (2). The brachial plexus is composed of the C5 to C8 cervical nerves and the anterior branch of the T1 thoracic nerve (3). Any nerve injury involving the brachial plexus is referred to be BPI. Excessive traction is one of the major mechanisms leading to BPI, which happened on 95% of BPI patients and even more (4). Previous studies have shown that surgical intervention is considered to be the final solution for clinical management of BPI, but it carries high risks and costs (5). In non-surgical treatments, local corticosteroid injections, splinting, and oral corticosteroids have shown significant short-term benefits but are prone to recurrence and may lead to complications such as mechanical or chemical nerve damage (6). Therefore, selecting the optimal treatment approach for BPI is crucial. Electroacupuncture (EA) for BPI has been proven to be clinically effective with less adverse reactions, low long-term recurrence rates, and is considered as the best conservative treatment option for patients who unwilling to undergo surgery (4, 7). Current cases mostly use electromyography, computed tomography (CT), and other examinations to evaluate BPI, but the full data of brachial plexus edema, pain, limb motor, and mood throughout the treatment is not reported yet.

Currently, CT myelography is the gold standard for diagnosing BPI. However, some patients are afraid of the side effects of CT myelography (such as radiation, bleeding, infection, low-pressure headaches, allergies, etc.), and therefore refuse this examination (8). Magnetic resonance imaging (MRI) is more expensive and takes a longer time to perform the examination, and the patient is needed to keep calm in order to obtain clear imaging. Similarly, ultrasound can be used to evaluate almost all peripheral neuropathies without any radiation damage. But it is of high quality, low price, timely, and effective. It can clearly observe the shape and movement of the brachial plexus nerves, and can locate nerve entrapment (9).

Although early diagnosis and treatment of BPI play a very important role in effectively improving the prognosis, unexpected situations often occur, such as delays and unexpected situations happening in treatment and aggravation of symptoms after some treatments (10). This article will present a case of BPI, in which the patient was injured twice after brachial plexus injury, and her symptoms worsened after undergoing rehabilitation and massage. The patient was assessed by the musculoskeletal ultrasound examination in different time points. Finally, she received EA to relieve his symptoms, and no recurrence happened after two years of follow-up.

## Case presentation

The patient was a 46-year-old female who experienced a strain in the left shoulder, resulting in difficulty in raising, flexing and extending of the left upper limb for 2 weeks. Subsequently, the patient's left shoulder pain worsened during dancing, and then she was diagnosed as BPI at the local hospital. Cervical spine X-ray in the lateral view revealed osteophyte formation at the C4-C6 vertebra and stenosis of the C4/5 intervertebral space. The patient received the rehabilitation and massage, but the symptoms worsened, and spontaneous twitching (Supplementary Video 1) in the left shoulder appeared. Further test revealed subacromial bursitis and a small cystic lesion in the proximal humerus of the left shoulder, as well as supraspinatus tendonitis on the ipsilateral side. As the condition gradually worsened, the patient was diagnosed with depression and anxiety symptoms, and accompanied by insomnia. As medications such as escitalopram, ibuprofen, and clonazepam were prescribed, the emotional disorder and insomnia of the patient were improved, while which didn't have significant effect on the pain and movement disorder (Table 2). In June 2020, the patient was admitted and the multi-disciplinary treatment was arranged for her.

After the physical examination, we found that the bilateral neck muscles were tense, which were accompanied with pain (Table 2) and restricted neck movement. Besides, the patient also appeared positive for paravertebral tenderness in the cervical 3–7 spinous process, positive for tenderness in the upper corner of the left scapula, and positive for left brachial plexus traction test. However, the results of both intervertebral foraminal compression test and neck rotation test are negative. The muscle tension of the limbs, muscle strength of the right upper limb, and tendon reflexes in both upper limbs are normal. The examination on muscle strength test of the left upper limb is uncooperative. Besides, the bilateral Hoffmann sign are negative.

**Figure 1:**
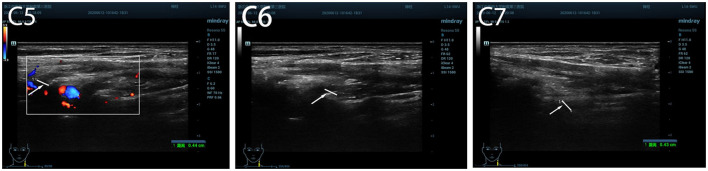
The inner diameter of cervical nerve root at C5, C6 C7 before treatment.

Musculoskeletal ultrasound of the brachial plexus showed an increased inner diameter at the left C5 to C7 nerve root outlet compared to the right side, with uneven internal echoes and enhanced nerve sheath echoes. The inner diameter at the C5 nerve root outlet was approximately 0.44 cm, at the C6 nerve root exit was approximately 0.46 cm, and at the C7 nerve root exit was ~0.43 cm (Figure 1).

In order to further confirm the diagnosis, the patient underwent other tests. All blood tests, including the routine blood test, blood biochemical, electrolyte and metabolic profiles, were normal. The result of CT on the brain suggested no evidence of intracranial hemorrhage or territory infarction. The MRI of left shoulder revealed that left-sided cystic degeneration of the upper end of the humerus and left supraspinatus tendon disease. The electromyography of left upper limb shows no abnormalities. Thus, the patient was clearly diagnosed with BPI.

This patient was treated with stainless steel acupuncture needles (size: 0.25 mm × 40 mm) inserted into a depth of approximately 1.5 cun. Acupuncture was performed at acupoints, namely Jiaji (EX-B2), Jianyu (LI15), Jianliao (SJ14), Binao (LI14), Hegu (LI4) and Waiguan (SJ5) (Table 1). Connect one pair of positive and negative electrodes of the electrical stimulation device to Jianyu (LI15) and Jianliao (SJ14), and connect the other pair of positive and negative electrodes to Jiaji (EX-B2) upper and lower points bilaterally (Supplementary Figure 1). Set the knob of the electrical stimulation device (HANS-200A) to zero, select mode of continuous wave with a frequency of 2 Hz (11). Then, turn on the power, adjust the intensity gradually until the patient can tolerate it, based on the occurrence of rhythmic muscle contractions locally. Each session lasts for 30 min. After treatment, turn off the power, remove the electrodes, and withdraw the needle. Treat once every 2 days, continuously for 3 months.

**Table 1:** The framework of the acupuncture point prescription.

**Acupoints**	**Locations**
Jiaji (EX-B2)	Bilateral side to the posterior midline of the cervical spine, 0.5 cun apart from the 1st to 7th cervical spinous process
Jianyu (LI15)	Anterior and inferior to the acromion, in the depression between the acromion and the greater tuberosity of the humerus
Jianliao (SJ14)	In a depression appears posterior and inferior to the acromion when the arm is abducted.
Binao (LI14)	On the outside of the arm, at the insertion point of the deltoid muscle, 7 cun above Quchi point
Hegu (LI4)	Between the first and second metacarpals, and at the midpoint of the radial side of the second metacarpal

**Figure 2:**
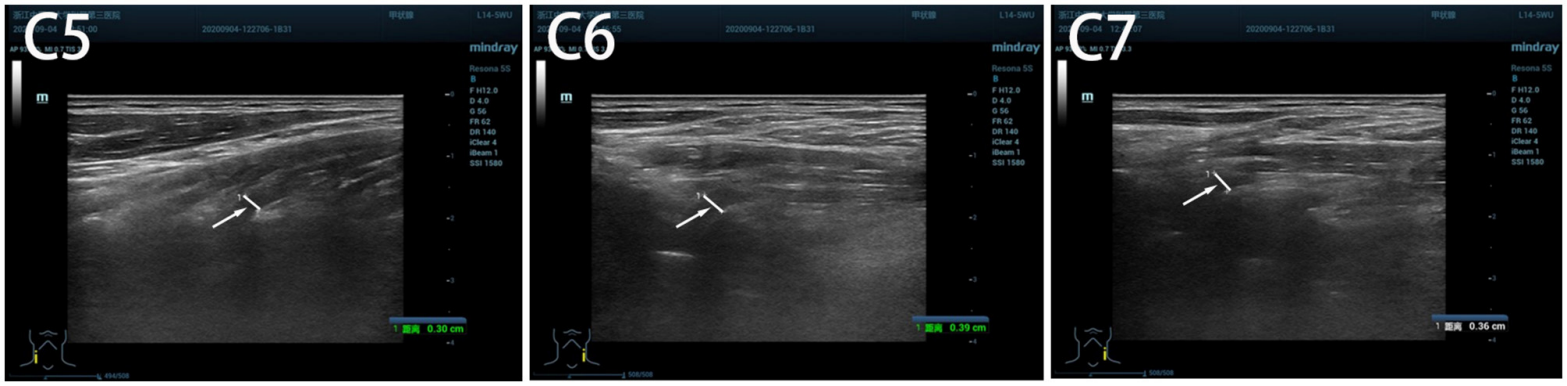
The inner diameter of cervical nerve root at C5, C6, C7 after 3 months' treatment.

After 3 months of EA treatment, the ultrasound of brachial plexus showed that the inner diameter of the left C5 to C7 nerve root exits was relatively wider than the right side, with uneven internal echoes and enhanced nerve sheath echoes. The inner diameter at the C5 nerve root exit on the left side was approximately 0.38 cm, at the C6 nerve root outlet was approximately 0.39 cm, and at the C7 nerve root outlet was approximately 0.36 cm (Figure 2). Compared with pre-treatment, the inner diameter of the left C5 to C7 brachial plexus nerve root outlet was significantly reduced, the pain of the patient's left neck and shoulder was significantly relieved (Table 2), and the movement disorder of limbs was improved (Table 2, Supplementary Video 2).

**Table 2:** The assessment of pain and mood.

**Time points**	**VAS**	**HAMA**	**HAMD**
Before admission	6	10	12
3 months after admission	2	8	9
2 years after treatment	0	7	6

**Figure 3:**
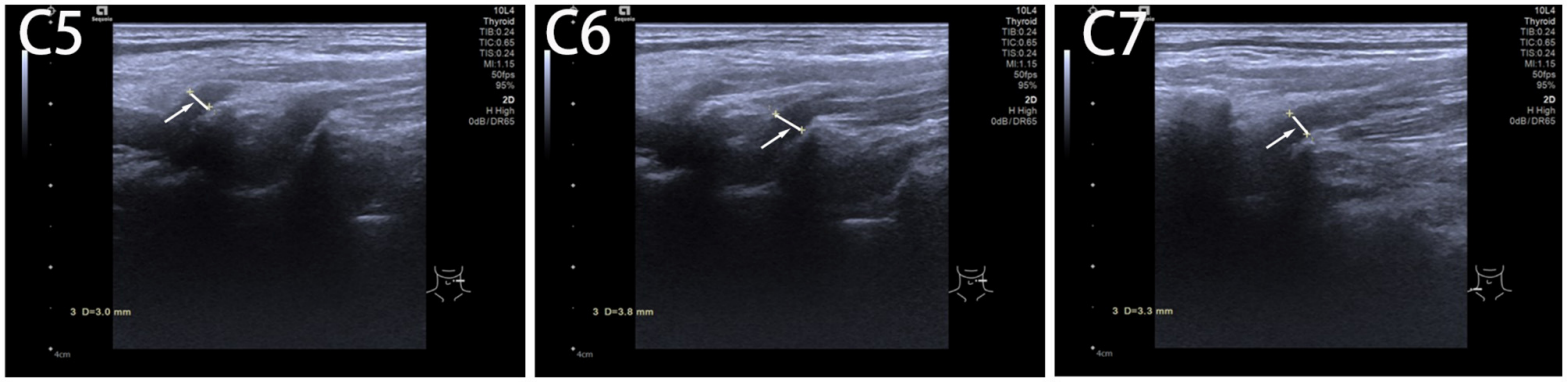
The inner diameter of cervical nerve root at C5, C6 C7 after 2 years of follow-up.

**Figure 4:**
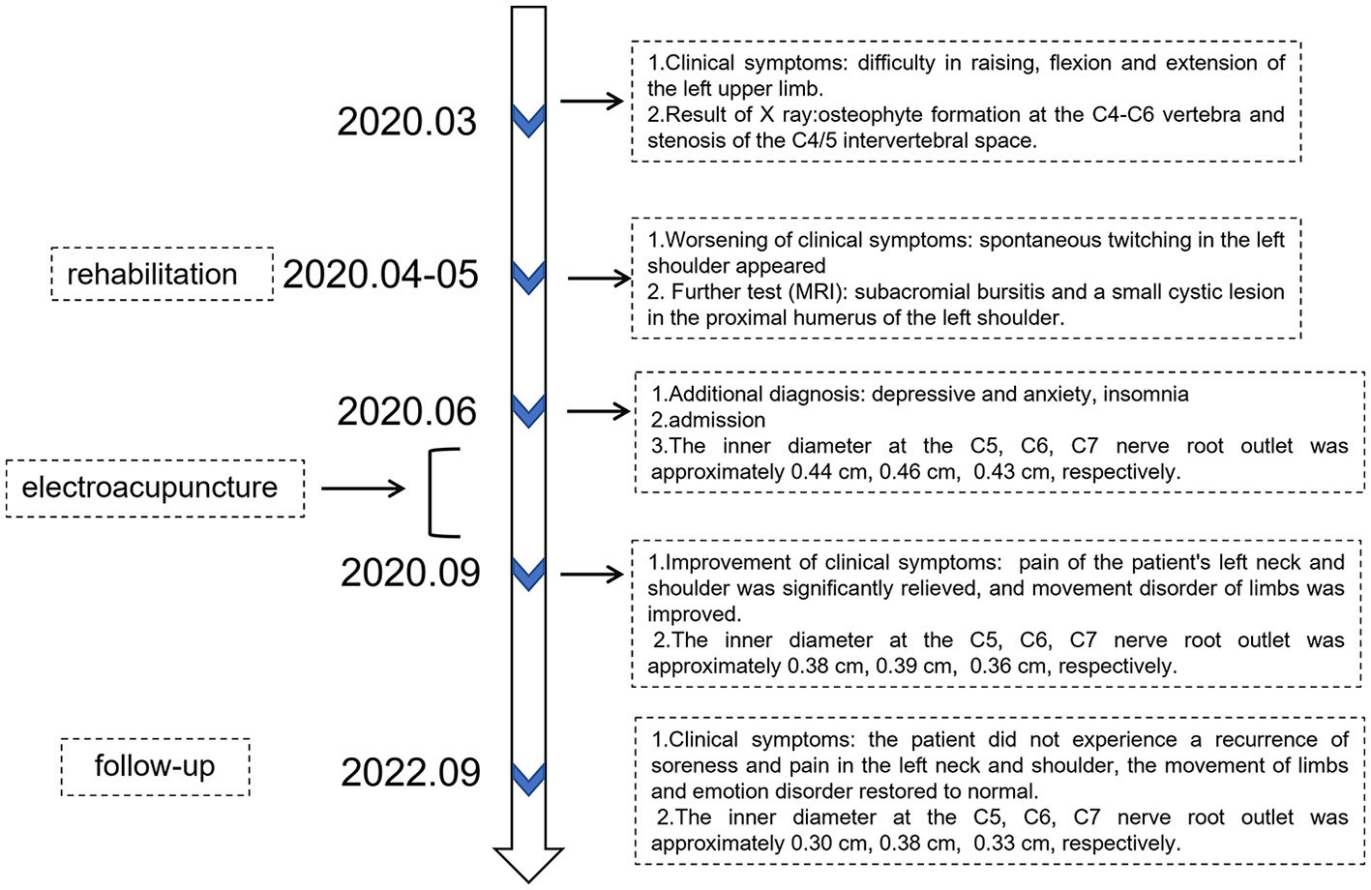
The timeline of disease course.

After 2 years of follow-up, it was found that the inner diameter of the left C5 to C7 nerve root outlet was relatively wider than the right side, with even internal echoes and normal nerve sheath echoes. The inner diameter at the C5 nerve root outlet on the left side was approximately 0.30 cm, at the C6 nerve root outlet was approximately 0.38 cm, and at the C7 nerve root outlet was approximately 0.33 cm (Figure 3). Compared with the musculoskeletal ultrasound on BPI patient 2 years ago, the inner diameter of the left C5 to C7 brachial plexus nerve root outlet narrowed, and the patient did not experience a recurrence of soreness and pain (Table 2) in the left neck and shoulder, and the movement of limbs and emotion disorder restored to normal (Tables 2, 3). The timeline of this case report is shown in Figure 4.

**Table 3:** The assessment on the range of shoulder motion.

**Time points**	**Forward flexion**	**Active abduction**	**Active external rotation**
Before admission	60	90	23
3 months after admission	125	130	75
2 years after treatment	145	143	83

## Discussion

EA is a treatment in Traditional Chinese Medicine (TCM) that combines acupuncture to electrotherapy (12, 13). It regulates the physiological functions and disease state of the body by applying electrical stimulation at specific acupuncture points. EA analgesia mainly involves in inhibiting afferent spinal cord signals, mediating the release of substances (such as TNF-α), participating in axonal reflexes and nerve impulse regulation, thereby reducing pain sensation (14). Furthermore, EA has been shown to improve the microenvironment of the injured nervous system by increasing levels of endogenous neurotrophic factors and reducing inflammation, thereby rebuilding neuronal circuits, restoring motor and sensory functions (15, 16). In addition, EA can reduce stroke-related nerve damage by promoting angiogenesis, alleviating inflammatory response, and regulating the blood-brain barrier (BBB) (17, 18). Some studies have proposed different mechanism of EA such as a decrease of C nerve fibers response in the spinal cord after repeated PNM in the sciatic or tibial nerve division in induced neuropathic nerves in cats (19). Although there is currently controversy over the mechanism of EA, the different EA parameters can produce a marked effect in specific situation. Clinical trials have shown improvements in strength, pain, and range of motion after the shoulder treated by EA (20, 21). The application of EA on neurogenic pain has shown that the ectopic emission signal of the damaged nerve is reduced, which translates into a reduction in pain perception (22), but evidence on this mechanism remains insufficient and further research is needed.

The BPI is one of the more serious peripheral nerve injury diseases, with partial or even complete loss sensorimotor function of the upper limbs as the main manifestation (23). The prevalence of the disease is increasing year by year, mainly caused by motor vehicle accidents, especially motorcycle traffic accidents (24) and traction injuries during neonatal delivery (25), which is seriously affecting people's daily life. There are a few case reports about BPI treated by acupuncture, but few papers are related to EA. In this case report, we observed the morphology of brachial plexus in different time points through musculoskeletal ultrasound which can directly reflect the damage and recovery of nerves. Meanwhile, we also estimated the pain and emotion of patient with BPI due the closed relationship between them. Different to previous reports, we have selected a middle-aged female patient who have experienced two traumatic injuries on brachial plexus, and their symptoms have worsened after other treatments. Thus, the process of their illness and changes are relatively complex. This case is about a 46-year-old female patient with brachial plexus damage and mixed cervical spondylosis, accompanied with poor lift, limited flexion and extension, spontaneous twitching of her left upper extremity, and pain in the left neck and shoulder treated with the EA. Musculoskeletal ultrasound in this case report revealed a significant reduction in the edema of brachial plexus nerve root. And the soreness, pain, and movement disorder in the left neck and shoulder were significantly reduced by the EA. During a two-year follow-up, the symptoms of BPI did not recur, and emotion disorder returned to normal. Based on the meridian theory and clinical practice experience of TCM, the acupuncture points such as Jiaji (EX-B2), Jianyu (LI15), Jianliao (SJ14), Binao (LI14), Hegu (LI4) and Waiguan (SJ5) were selected according to the local and remote therapeutic effect in the therapy of acupuncture. These acupuncture points are located in the distribution area of brachial plexus, blood vessels and tissues. The electrostimulation on these points can increase local blood circulation, repair nerve conduction (26, 27), and promote tissue repair. Meanwhile, EA is proved to decrease the edema of nerve (28) that is as same as what reported in this case report. The strength of the EA is the satisfactory effect on pain and motion which can last for 2 years without recurrence. Moreover, it is worth noting that the patient experienced local subcutaneous bleeding during the treatment, but the patient agreed to continue treatment only after communicating with the patient.

However, there were some limitations in this case report. Firstly, due to the number of cases involved in this case report is merely one, the evidence of the EA effectiveness is one-sided. Secondly, this case is under a specific situation, and no general conclusions can be drawn. Besides, the ultrasonic system and ultrasound technician in this report was changed within 2 years, which may lead to some biased results. In addition, since the patient had been taking analgesics and mood drugs, the impact of the medication on BPI patients could not be thoroughly excluded.

The musculoskeletal ultrasound, as one of the assessment methods, can provide more objective clinical evidence for the promotion of EA in future research. However, musculoskeletal ultrasound is still a maneuver-dependent tool, repeated measurement must be conducted to avoid bias. The current treatment duration and optimal parameters for EA treatment of BPI still require more in-depth studies to elucidate.

## Conclusions

After 3 months of EA treatment, pain and joint function are improved for patients with BPI. And after 2 years, the patient did not experience a recurrence of soreness and pain in the left neck and shoulder. We understand that this study focuses only on the changes in the clinical symptom and musculoskeletal ultrasound, the bias of the results still can't be avoided. We hope that this case report can provide some theoretical bases for further exploring the clinical evidence of EA for BPI in the future.

## Data availability statement

The original contributions presented in the study are included in the article/Supplementary material, further inquiries can be directed to the corresponding authors.

## Ethics statement

The studies involving humans were approved by Third Affiliated Hospital of Zhejiang Chinese Medical University. The studies were conducted in accordance with the local legislation and institutional requirements. The participants provided their written informed consent to participate in this study. Written informed consent was obtained from the individual(s) for the publication of any potentially identifiable images or data included in this article.

## Author contributions

CW: Writing – original draft. YL: Writing – original draft. LL: Methodology, Writing – review & editing. HZ: Writing – original draft. ZY: Investigation, Writing – review & editing. LZ: Conceptualization, Writing – review & editing.
