# Supplementary figures and images for: The efficacy of electroacupuncture for cervical nerve edema and movement disorder caused by the brachial plexus injury: a case report
Source: Front Neurol. 2024 Apr 23;15:1342844. doi: 10.3389/fneur.2024.1342844 (PMC11075755; doi:10.3389/fneur.2024.1342844)

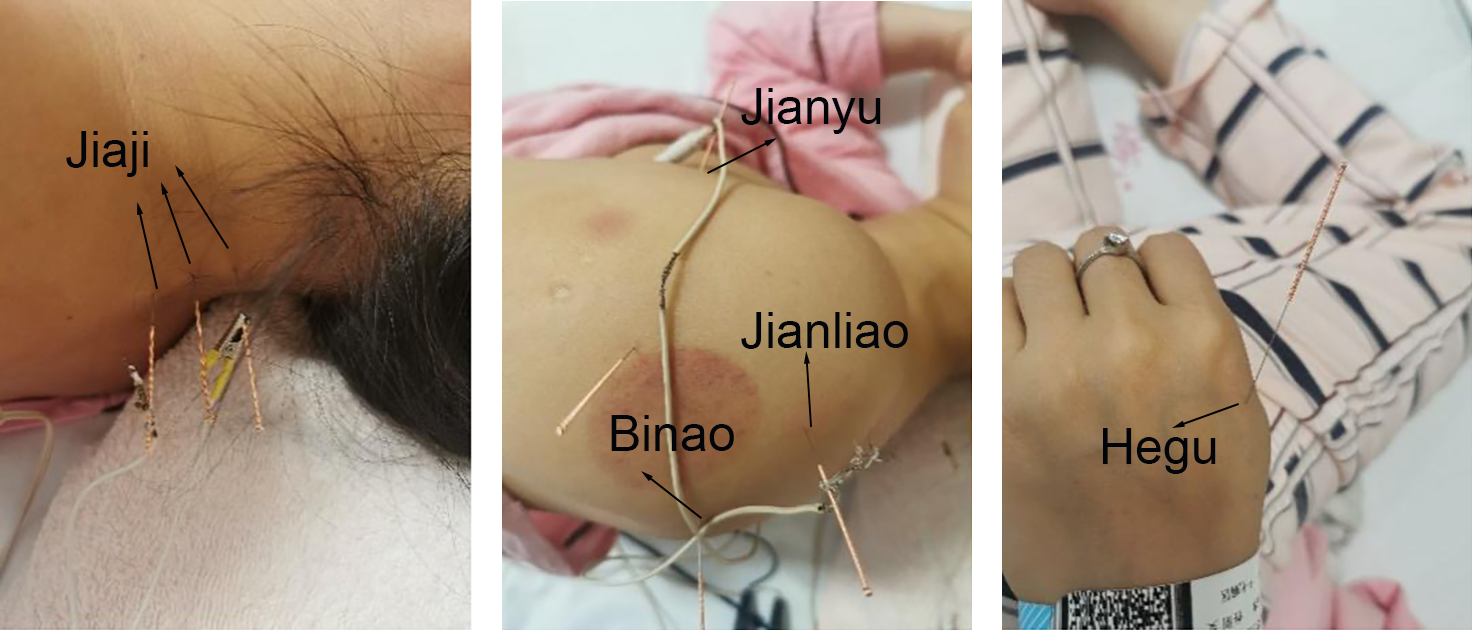

Supplement: Supplementary file 3 [file Image_1.TIF]
